# EZB-ICR Cell Line: A New Established and Characterized Oral Squamous Cell Carcinoma Cell Line From Tongue

**DOI:** 10.31557/APJCP.2021.22.1.99

**Published:** 2021-01

**Authors:** Nooshafarin Chenari, Bijan Khademi, Mahboobeh Razmkhah

**Affiliations:** 1 *Institute for Cancer Research, School of Medicine, Shiraz University of Medical Sciences, Shiraz, Iran. *; 2 *Research Center of Otolaryngology Head and Neck Surgery, Shiraz University of Medical Sciences, Shiraz, Iran. *; 3 *Department of Otolaryngology, School of Medicine, Shiraz University of Medical Sciences,Shiraz, Iran. *

**Keywords:** Tongue cancer, oral squamous cell carcinoma, EZB-ICR, head and neck cancer

## Abstract

**Background:**

Tongue cancer is one of the most aggressive forms of oral squamous cell carcinoma which needs more investigations. Herein, we aimed to establish and characterize a tongue cancer cell line.

**Methods:**

Tumor tissue was obtained from a 70-year-old woman with tongue cancer. The established cell line named as EZB-ICR and characterized for doubling time, expression of specific markers, HPV corporation and migration status using flow cytometry, immunofluorescence staining, multiplex PCR, and migration assay.

**Results:**

EZB-ICR was negative for expression of mesenchymal specific markers, cytokeratin19, pan-cytokeratin, vimentin and EPCAM, but was positive for S100 and Nestin. No appearance of human papilloma virus DNA was seen. The doubling time of EZB-ICR was 31 hours and migration assay confirmed its metastatic nature.

**Conclusion:**

To the best of our knowledge, EZB-ICR is the first tongue human cancer cell line established in Iran, and its features make it appropriate for cancer-based in vitro studies and contribute to more studies on tongue cancer.

## Introduction

Head and neck cancer is the world’s fifth most common type of cancer worldwide. Oral squamous cell carcinoma (OSCC) is a prevalent cancer consisted of more than 90% of oral malignancies and in spite of recent advancements in diagnostic approaches and treatments, 5 year survival showed no difference in recent years (Sinevici and O’sullivan, 2016; Bagan et al., 2010). The most common oral squamous cell carcinoma is found in tongue and mouth floor (Hamiter et al., 2017). The age range of these patients is 20–44 years old and consuming tobacco and alcohol are among the most important risk factors. Furthermore, genetic, environmental or viral factors may lead to the pathogenesis of tongue cancer (Patel et al., 2011).The feature of tongue cancer is completely different from malignancies of other parts of oral cavity because tongue has a significant lymphatic structure and as a result shows aggressive metastatic pattern (Lim, 2006). Based on the American Cancer Society, 5-year relative survival rate for tongue cancer is as follows: 81% for localized (no sign the cancer has spread outside), 68% for regional (spread to nearby structures or lymph nodes), and 39% for distant (spread to distant parts of the body) type of tongue cancer (American Cancer Society, cancer.org |1.800.227.2345). 

Although tongue cancer is increasing throughout the world, no effective treatment is exist and common therapies such as surgery, radiation therapy, chemotherapy and combination therapies are not enough (Zhong et al., 2019). Thus more in vitro and in vivo investigations using tongue cancer cell lines are undoubtedly needed to establish new therapeutic intervention for tongue cancer. Here, we established a new tongue cancer cell line named EZB-ICR and characterized it for the expression of various markers, growth condition, HPV status and metastatic pattern.

## Materials and Methods

*Cell line establishment*

In this experimental study, tumor tissue sample was obtained from a 70 years old woman patient with tongue cancer. The fragments were washed with sterile phosphate buffered saline (PBS) containing 1% pencillin/streptomycin. Next, the segments were cultured as a transplantation method in complete DMEM (containing 10% fetal bovine serum (FBS) and 1% penicillin/streptomycin) at 37°C in a humidified atmosphere containing 5% CO_2_. The media was changed every 48 hours. 

*Growth curve and proliferation assay*

For evaluation of doubling time and growth rate of the cell line established, 2.5 × 10^4^ cells were seeded on six well tissue culture plates and the number of the cells was calculated each day for 7 days; this experiment was repeated three times. Growth curve and doubling time were measured by online doubling time calculating software (http://www.doubling-time.com).

*Flow cytometry*

For phenotypical characterization, the cells were trypsinized and washed with PBS. Then the cells were stained with the appropriate flourchrome labeled monoclonal antibodies against mesenchymal lineage markers such as anti-CD34, CD19, CD166, CD105, CD44, CD10, CD146, CD29, CD45, CD73, CD90, CD24, CD69 and also some chemokine/chemokine receptors like MCP1, IP-10, RANTES, EPCAM and CXCR4, CCR5. The cells were fixed by 1% cell fix for 10 minutes and then washed with PBS. Next, 500ml of 0.2% Saponin was added, and incubated for 15 minutes and washed again. Consequently, cells were stained with above mentioned antibodies (All from BD Bioscience, USA) and finally were evaluated using FACSculibure flow cytometer and analyzed by FlowJo software version 10.

*Immunofluorescent assay*

Nestin, cytokeratin 19, vimentin, pancytokeratin and S100 were assessed with immunofluorescent assay using appropriate antibodies (all from Santa Cruz and Abcam). 3 × 10^4^ cells were seeded on chamber slides for 24 hours. Then, the slides were washed by PBS and fixed with cold methanol at room temperature, and blocked with 1% bovine serum albumin (BSA) in PBS containing 1% Triton X-100 (PBST). Subsequently, slides were incubated with primary monoclonal antibodies overnight at 4°C in 1.5% BSA in PBST. Then, slides were washed with PBST and incubated with secondary fluorescent antibodies (goat anti-mouse IgG- conjugated with FITC, Santa Cruz) for 2 hours at room temperature. After that, the slides were mounted by solution containing propidium iodide (PI) and observed by fluorescent microscope. Our negative controls were the slides without antibodies and those treated with secondary antibody. 

*Migration assay*

The motility of cells was investigated by migration assay via transwell inserts. 5×10^3^ cells were cultured in the upper 8 μm transwell cell culture chambers in the FBS deficient condition media and the lower wells was filled out by 650 μl of DMEM media containing 10% FBS. The cells were incubated at 37 °C in 5% CO_2_ humidified chamber for 24 hours. Then, the non-migratory cells were eliminated from the upper side by cotton swab and the cells which were transferred from upper to lower chamber were fixed and stained with 10% ethanol and 0.5% Crystal Violet. At the end, the migrated cells were counted by phase contrast microscope. This assay was performed in triplicate for each cell line including EZB-ICR, KB (as one source of mouth epidermal carcinoma) and MDA-MB-231 (as an invasive cell line). 

*Colony formation unit assay (CFU)*

The EZB-ICR cells were harvested with trypsin–EDTA and around 1,000 single cells were seeded in 25mm tissue culture flask in DMEM containing 10% FBS and 1% penicillin/streptomycin. On the 5^th^ and 10^th^ days of culture, cells were washed with PBS and stained by 0.5% Crystal Violet in methanol for 15 minutes at room temperature, finally flasks were washed by PBS and colonies were calculated by following formulation: colony number/cells of seeding ×100.

*Human Papilloma Virus (HPV) molecular genotyping (32 genotypes)*

Total DNA of EZB-ICR cells was extracted by fully automated instrument, part of SPF10 region of the HPV genome was amplified using Multiplex PCR method and the genotype of HPV was determined by various specific probes using reverse hybridization.

**Table 1:** The Percentage of Markers in EZB-ICR Cell Line

Marker	CD10	CD90	CD29	CD146	CD166	EPCAM	CD14	CD24	CD34	CD44
Percentage (%)	0.023	0.01	0.015	0.092	0.021	0.015	0.017	0.061	0.013	0.015
Marker	CD45	CD105	CD73	CD106	RANTES	MCP1	CD69	CXCR4	CCR5	
Percentage (%)	0.013	0.037	0.037	0.023	0.14	0.19	1.12	0.2	0.028	

**Figure 1:**
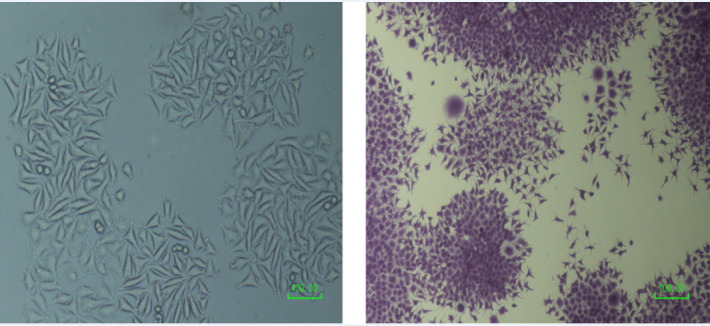
Cellular Morphology of EZB-ICR Cell Line in Cell Culture. EZB-ICR was appeared as polygonal in shape with regular dimensions

**Figure 2:**
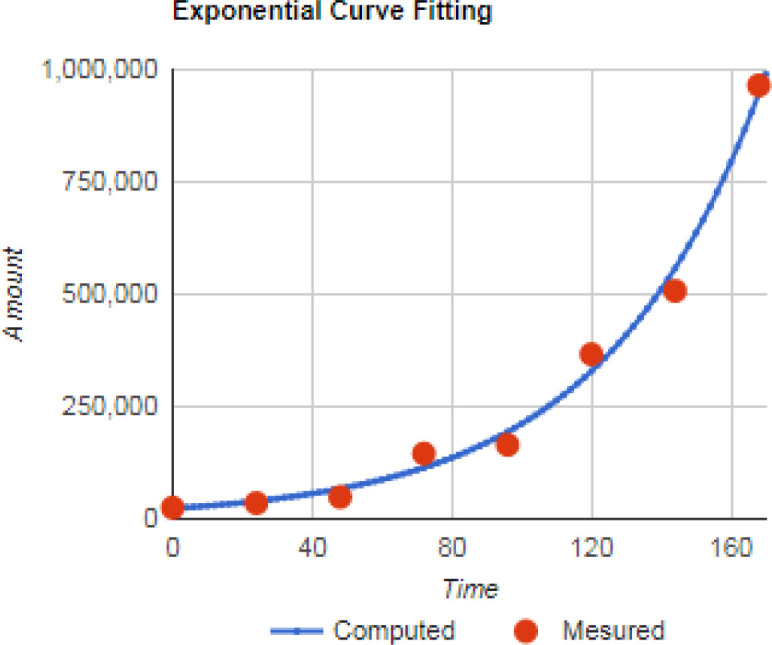
Growth Curve of EZB-ICR for 7 Days. The doubling time of EZB-ICR was about 31 hours

**Figure 3:**
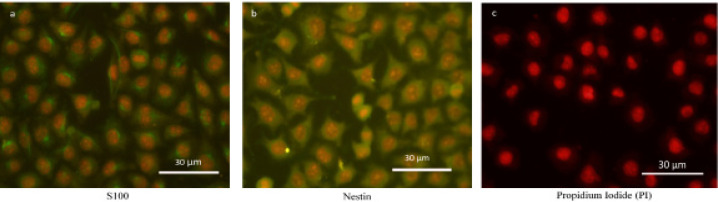
Immunofluorescence Staining of EZB-ICR Cell Line. Expression of S100 (a) and Nestin (b) in EZB-ICR showed positivity for these two markers. The nucleus was stained by propidium iodide (PI) (c).

**Figure 4:**
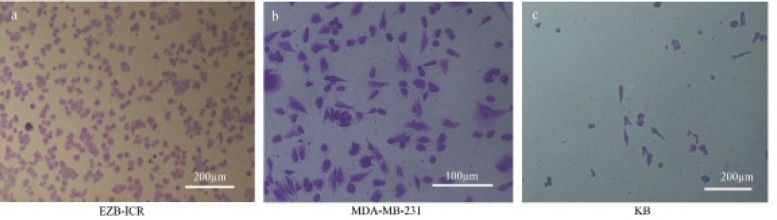
Migration Assay for EZB-ICR Cell Line Compared to MDA-MB-231 and KB Cell Lines. EZB-ICR cell line (a) showed higher capability of migration compared to MDA-MB-231 (b) and in particular KB (c) cell lines

**Figure 5:**
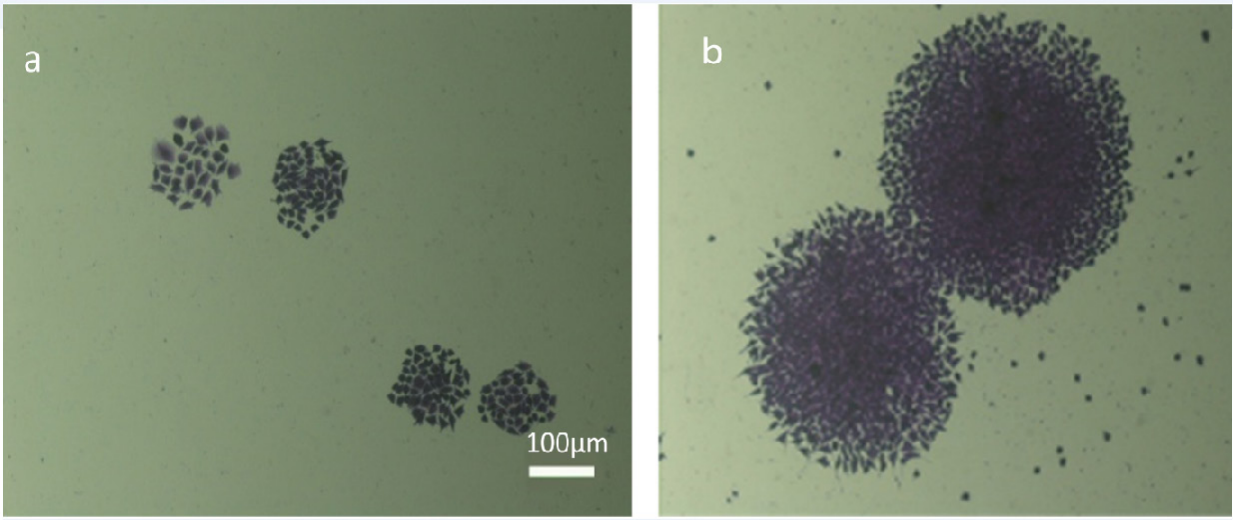
Colony Formation Assay, 5 days (a) and 10 days (b) post culture of EZB-ICR cell line showed the capability of this cell line for forming distinct colonies

## Results

*Establishing and characterizing tongue cancer cell line*

EZB-ICR cell line was generated from a 70-year-old woman patient that used waterpipe (hubble-bubble) sometimes. Examination diagnosed aggressive squamous cell carcinoma in right oral tongue lesion in 2016. The tumor mass size was 2.7x1.8x 0.7 cm at right lateroinferior site with ulcerative surface and also, base, apex and medial margins grossly not involved and inferior margin grossly seems to be involved. The patient underwent a hemiglossectomy surgery. The cells were firstly extracted as explants of chopped tumor mass and the cells were grown out after 3 weeks. EZB-ICR cell line was passaged frequently until 72 passages (Figure 1).

*Doubling time*

Doubling time of EZB-ICR cell line was assessed at passage 25 in the course of the exponential growth phase of the cell line by calculating the number of cells for 7 days. Accordingly, the doubling time of EZB-ICR was about 31 hours (Figure 2).

*Flow cytometry*

Flow cytometry examination indicated that EZB-ICR cell line was negative for mesenchymal stem cell markers such as CD166, CD44, CD90,CD146 and also markers such as CD10, CD45, CD34, CD105, CD106, CD14,CD24, CD29, CCR5, CD69, CXCR4, IP10, MCP-1, RANTES and EPCAM as depicted in Table 1.

*Immunostaining*

According to immunofluorescent staining assay, EZB-ICR cell line was strongly positive for the expression of S100 and nestin but it was negative for cytokeratin19, pan-cytokeratin and vimentin (Figure 3).

*HPV molecular genotyping*

EZB-ICR cell line was negative for 32 genotypes of HPV. These genotypes were divided to 4 subgroups which included high risk (16, 18, 31, 33, 35, 39, 45, 51, 52, 56, 58, 59, 68), partially high risk (26, 53, 66, 70, 73, 82), Low risk (6,11,40,42,43,44,54,61,81) and finally unclassified HPV genotypes (62, 67, 83, 89). 

*Migration assay*

Metastatic ability of EZB-ICR cell line was evaluated by migration assay and compared with MDA-MB-231 breast cancer cell line as an invasive cell line and KB cell line which originally from epidermal carcinoma of the mouth. EZB-ICR showed higher capability of migration compared to MDA-MB-231 and in particular KB cell lines (Figure 4).

*Colony formation assay*

The colony formation assay was applied to recognize the ability of the EZB -ICR cell line to grow into a colony forming shape. In this regard the number of colonies after 5 and 10 days were respectively 63 and 45% (Figure 5).

## Discussion

Establishment of cell line is a profitable option for further understanding the pathways which contribute to the development of models for cancer metastasis and invasion. In addition, cell lines are required for evaluating the effects of numerous novel drugs and finding better treatments (Kaur and Ralhan, 2003). Several studies identified that the wide variety of patients who suffered from tongue cancer were smokers and most cell lines were determined from them, whereas few cell lines recognized from tumors of nonsmokers (Patil et al., 2014).

In the present study, we established and characterized the tumorigenic and metastatic EZB-ICR tongue cell line. EZB-ICR cell line did not express any surface mesenchymal markers. Also, it was not positive for the expression of cytokeratin19, pan-cytokeratin and vimentin. In contrast, this cell line was positive for the expression of S100 as well as nestin.

It has been recently demonstrated that S100 plays significant roles in biological processes such as proliferation, apoptosis and cell survival associated with both normal development and tumorigenesis (Donato, 2003; Donato et al., 2017). *In vivo* and* in vitro* experimental studies showed the over-expression of one subgroup of S100, S100A4, related to potential metastatic and poor clinical outcomes in different kinds of cancers (Chen et al., 2014). Experimental studies demonstrated the expression of nestin in several tumor cells like gastrointestinal stromal tumors, malignant melanoma, OSCC, pancreatic, prostate and breast cancers (Ishiwata et al., 2011). High expression of nestin correlates with consistent growth and poor treatment prognosis in most solid tumors (Singh et al., 2019). As our established cell line expresses both S100 and nestin, these markers may be considered as tongue tumor markers for diagnostic and therapeutic options.

Wanga et al., (2017) established a tongue cancer cell line from a nonsmoker 44-year-old woman (UCSF-OT-1109). Experimental studies demonstrated that never-smoking patients had aggressive patterns and their survival were even less than smoker patients (Durr et al., 2013; Wang et al., 2017). EZB-ICR and UCSF-OT-1109 both were isolated from nonsmoker patients and showed similar morphology. Although the connection of HPV infection to OSCC has been hypothesized(Gupta and Gupta, 2015), it did not emerge in neither EZB-ICR nor UCSF-OT-1109 (Wang et al., 2017). In contrast to EZB-ICR that did not express CD44 and EpCAM, UCSF-OT-1109 was positive for both markers (Wang et al., 2017).

Migration index showed that EZB-ICR has strong motility in comparison with MDA-MB-231 and KB cell lines. So, this behavior could answer important questions regarding the metastatic pattern of the EZB-ICR cell line.

To the best of our knowledge, EZB -ICR is the first human tongue cancer cell line that registered in the Cell Bank of Pasteur Institute of Iran. Furthermore, the EZB-ICR has enabled us to figure out more about the characteristics of tongue cancer, consequently, enhance our information in regard to finding novel therapeutic possibilities. The key important limitation in this study was that the number of tongue cancer cell lines in Iran is rare so we did not have a chance to compare EZB-ICR with other tongue cancer cell lines.
